# Predictive Intelligence for Cholera in Ukraine?

**DOI:** 10.1029/2022GH000681

**Published:** 2022-09-01

**Authors:** Moiz Usmani, Kyle D. Brumfield, Bailey M. Magers, Anwar Huq, Rosa Barciela, Thanh H. Nguyen, Rita R. Colwell, Antarpreet Jutla

**Affiliations:** ^1^ GeoHealth and Hydrology Laboratory Department of Environmental Engineering Sciences University of Florida Gainesville FL USA; ^2^ Maryland Pathogen Research Institute University of Maryland College Park MD USA; ^3^ University of Maryland Institute for Advanced Computer Studies University of Maryland College Park MD USA; ^4^ Meteorological Office Exeter UK; ^5^ Department of Civil and Environmental Engineering University of Illinois at Urbana‐Champaign Urbana IL USA

**Keywords:** cholera, Ukraine, infectious disease, climate, environmental, vibrios

## Abstract

Cholera, an ancient waterborne diarrheal disease, remains a threat to public health, especially when climate/weather processes, microbiological parameters, and sociological determinants intersect with population vulnerabilities of loss of access to safe drinking water and sanitation infrastructure. The ongoing war in Ukraine has either damaged or severely crippled civil infrastructure, following which the human population is at risk of health disasters. This editorial highlights a perspective on using predictive intelligence to combat potential (and perhaps impending) cholera outbreaks in various regions of Ukraine. Reliable and judicious use of existing earth observations inspired mathematical algorithms integrating heuristic understanding of microbiological, sociological, and weather parameters have the potential to save or reduce the disease burden.

## Key Points

- The risk of cholera in Ukraine will become significant with the noteworthy presence of plankton in the coastal regions, along with anomalous warm air temperatures and heavy precipitation
- Earth observations inspired anticipatory decision‐making framework is needed for preventing infectious disease outbreaks such as cholera in human population
- Regions such as Pakistan with massive flooding may experience similar risks and should be evaluated accordingly

## Editorial

Cholera, a deadly waterborne diarrheal disease, is generally associated with poverty and severe lack of hygiene, access to safe drinking water, and sanitation infrastructure. As such, the disease is frequently reported in regions where environmental, weather/climate, and societal vulnerabilities intersect with the bacterium, *Vibrio cholerae*, the causative agent, in the human population. The disease surfaced in both Africa and Europe in the 1970s. Between 1970 and 2011, several European countries reported outbreaks of a few to more than 2,000 cases (Oprea et al., 2020), mainly attributable to civil unrest. A prime example is Romania, where no cholera cases were reported to the World Health Organization before 1989. However, due to the unstable political situation following the Romanian Revolution, over 700 cases were reported between 1990 and 1995 (WHO, 2017 ). Between 1991 and 2011, Ukraine reported cholera outbreaks with nearly 1,500 cases, including at least 40 deaths (Oprea et al., 2020). More recently, Haiti (in 2010) and Yemen (2016) reported cholera epidemics totaling about 2.5 million cholera cases. In both of these regions, natural (earthquake in Haiti) and anthropogenic (civil unrest in Yemen) disasters severely damaged water, sanitation, and hygiene (WASH) infrastructure (Camacho et al., 2018; Hasan et al., 2012).

The Russian‐Ukrainian war has significantly impacted the world economy, geopolitics, food/water security, and the environment (details in Pereira et al. (2022), p.). Ongoing warfare is likely to devastate water availability and quality (Schillinger et al., 2020). Destruction of WASH infrastructure and lack of functioning drinking water facilities can trigger the spread of pathogens, as observed through prior civil conflicts, for example, Yemen. Moreover, pollutants of both biological and chemical origin transported from war‐affected areas can impact groundwater and marine ecosystems (Pereira et al., 2020). While the environmental impact of the Russian‐Ukrainian war is not yet fully understood, evidence is emerging that cities such as Mariupol and Chernihiv may be experiencing water shortages and deteriorated sanitary conditions (Pereira et al., 2022). It is also worth noting that clinically significant vibrios, particularly *V*. *cholerae*, have been recovered from coastal areas and inland regions of the warm subtropical climate of the Black Sea (Haley et al., 2014; Kokashvili et al., 2015; Terzi Gulel & Martinez‐Urtaza, 2016), with the highest detection rates in July, August, and September. However, ground surveillance of pathogens in war‐affected areas' aquatic environments is not always possible. Therefore, data‐rich heuristic algorithms using satellite sensors can provide predictive risk intelligence, a viable option for portending early warning of the environmental outbreak of diseases such as cholera in Ukraine.

Over the past 50 years, research has shown that *Vibrio cholerae* is native to the aquatic environment, where it proliferates when conditions are optimal (Choopun et al., 2002; R. R. Colwell & Spira, 1992; West & Lee, 1982; Xu et al., 1982). The growth and proliferation of *V*. *cholerae* and other *Vibrio spp*. are modulated by environmental parameters, namely ambient weather and climatic processes. For example, it is now known that coastal waters provide an ecological niche for pathogenic *Vibrio spp*., including *Vibrio parahaemolyticus*, *Vibrio vulnificus*, and *V. cholerae* (Brumfield et al., 2021). Furthermore, *Vibrio spp*. are commensal to copepods, a zooplankton comprising a significant component of aquatic fauna that feeds on phytoplankton in coastal waters (Tamplin et al., 1990). In fact, copepods are a major host of *V*. *cholerae*, sufficiently significant to be considered a vector (Brumfield et al., 2021). In summary, vibrios in the environment are strongly associated with the ecological and climate/weather processes, such as flooding (Khan et al., 2019; Koelle et al., 2005), sea surface temperature (Constantin de Magny et al., 2008; Lobitz et al., 2000), zooplankton (Huq et al., 1983, 1996), phytoplankton (R. Colwell & Huq, 2001); salinity (Miller et al., 1982); regional hydrology such as river flows (Akanda et al., 2009), coastal ecological processes such as planktonic growths (A. Jutla et al., 2013), ambient temperature (Speelmon et al., 2000), and precipitation (Hashizume et al., 2008; Pascual et al., 2002).

Cholera outbreaks generally occur in two modes (Codeço, 2001; Khan et al., 2017, 2018; Usmani et al., 2021): epidemic, namely a sudden occurrence of cholera in a region where a considerable disturbance occurs that causes interference with access to drinking water and proper sanitation and the human population is not prepared to deal with the disease; and endemic, a continuous occurrence of sporadic cholera cases with quasi‐predictable seasonality. The cholera epidemic mode can become endemic if safe drinking water access is not ensured. A cholera outbreak requires distinct trigger and transmission mechanisms (A. S. Jutla et al., 2010; Usmani et al., 2021). The trigger is defined as conditions that start (or initiate) an outbreak with environmental dynamics and a few cases in humans, and the transmission mechanism involves spreading the infection into human communities. While the origins of the cholera trigger continue to be debated (Khan et al., 2017), the interaction of humans with the environmental reservoirs of pathogenic vibrios is associated with outbreaks (Alam et al., 2006; Huq et al., 1983; A. Jutla et al., 2013; Singleton et al., 1982).

We emphasize that cholera is preventable by ensuring access to safe drinking water and sanitation, education, and ready availability of appropriate medicines. However, the key challenge is determining when and where to provide resources to prevent an epidemic outbreak. One solution is using predictive intelligence, a combination of field surveillance and outputs from mathematical models. Our previous research (A. Jutla et al., 2013), supported by several studies (A. Jutla et al., 2013, 2015; Khan et al., 2017, 2018; Lobitz et al., 2000), suggests that predictive intelligence can be developed to determine the likelihood of cholera risk in a particular region. Using historical data from the 1800s in India, a climate‐driven sociological hypothesis was developed that states that if a particular region experiences above‐average air temperatures, followed by heavy precipitation with considerable damage to water and sanitation infrastructure, that can change human behavior with respect to the consumption of water, then that region is at high risk of a cholera outbreak (details of the model are provided in a previously published study (A. Jutla et al., 2013, 2015)). If any of these conditions are not met, the outbreak potential for cholera will be low. A data driven score‐based mathematical algorithm developed over the last decade provides a reliable lead time of 4 weeks for cholera risk (Barciela et al., 2021; Khan et al., 2017, 2018) (https://vibrio-prediction-ufl.hub.arcgis.com/). This algorithm is able to provide outputs of risk values (high, medium, and low) at a 1 × 1 km pixel scale. It employs earth observations, including precipitation, temperature, population density, sociological factors such as access to drinking water and sanitation access, and microbiology‐based vibrio growth curves. The outputs of this algorithm and, therefore, the hypothesis is validated using datasets from various parts of Africa (A. Jutla et al., 2015), Nepal (Khan et al., 2018), Haiti (Khan et al., 2017), and more recently Yemen (Barciela et al., 2021). Yemen provided one of the first (and rare) opportunities to validate the model on a near‐real‐time (Barciela et al., 2021) basis. Currently, model accuracy is between 60% and 80% for the trigger component, with ca. four weeks of lead time from the date the forecasts are made.

**Figure 1:**
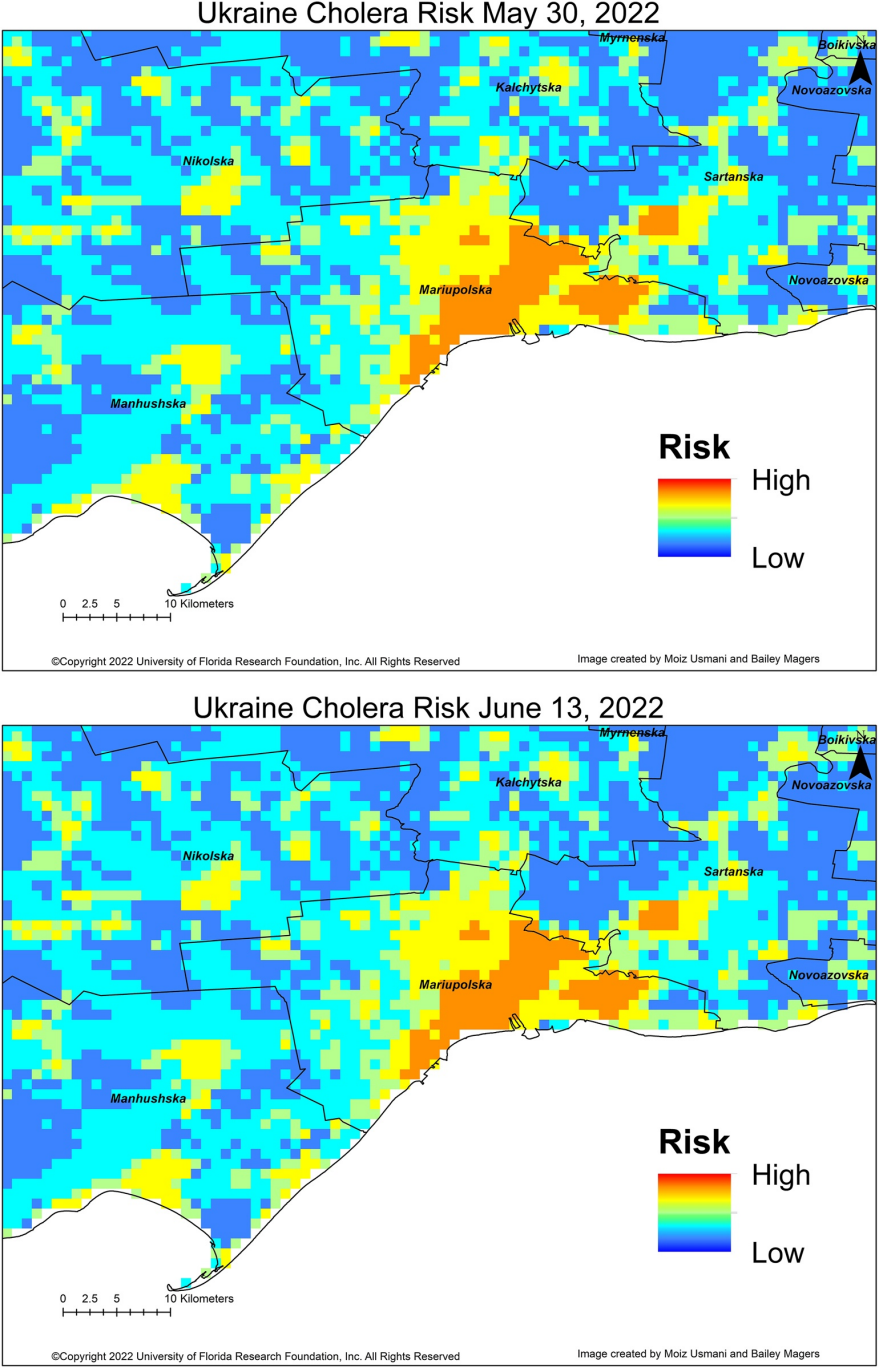
Cholera Risk on (a) 30 May 2022, and (b) latest prediction produced on 13 June 2022.

The cholera algorithm was implemented in Ukraine in May 2022, noting the loss of civil infrastructure, including water facilities and sanitation provisions, because of the conflict underway (English, 2022). Thus, if there are anomalous warm air temperatures and heavy precipitation, with the noteworthy presence of plankton in the coastal regions, the risk of cholera in Ukraine will become significant. Figure 1a shows the first set of data obtained since the war in Ukraine started, with the coastal region of Mariupol indicating a medium risk of cholera. Medium risk of cholera implies that if conditions become amplified (in this case, heavy rainfall), the region can expect to experience a cholera outbreak within the next 4 weeks of the forecast. Figure 1b shows the risk of cholera in the Mariupol region as of 13 June 2022, which now remains consistently at medium risk.

Geophysical processes employed to understand and develop predictive intelligence comprise a relatively new domain; therefore, it is expected that variation in scientific reasoning will occur. However, a holistic data‐driven adaptive understanding of pathogens whose life cycle is influenced by weather and climatic processes must be a part of decision‐making processes when anticipatory intervention actions are to be sought. In this regard, we clearly distinguish between the reactive and anticipatory decision‐making framework. Most decision‐making for infectious disease outbreaks remains reactionary, implying that intervention and mitigation efforts start after an outbreak is reported in a country or a region. Earth observations, sociological processes, and microbiology provide a unique opportunity to develop an anticipatory decision‐making framework for impending disease outbreaks (in this case, cholera). Ukraine has not yet reported a widespread cholera outbreak (as of 07/31/2022). However, given the complexities of the region where an appropriate diagnosis and population movements is perhaps not possible, key anticipatory intervention steps to stop a potential outbreak (or limit spread) must include (a) distribution of water safety kits, (b) stockpiling and ensuring availability of antibiotics, (c) provisions for timely vaccinations and (d) strengthening the education of the local population to ensure caution on handling water in conflicted regions. Further, it is worthwhile to highlight the importance of the collection of environmental samples of water from various locations (ponds, rivers, etc.), and such samples should be analyzed to ascertain the presence, abundance, and prevalence of pathogenic vibrios. In addition, the surveillance system for reporting diarrhea may be implemented in regions with access to the internet or data transmission. The spread of cholera from a particular region can be limited if the movement of the human population remains low, given that appropriate safeguards are provided in that region to save human lives. Preemptive actions that may be useful for avoiding a large‐scale cholera outbreak is shown are summarized in Table 1.

**Table 1:** Recommended Preemptive Actions

	Preemptive interventions	Preference	Source
Safe water	Sealed and bottled water	1	McLennan (2016)
	Water treatment	2	Sinyange et al. (2018), Taylor et al. (2015)
	Boiling water	3	Lantagne and Yates (2018)
Safe defecation	Limit open defecation	1	Cowman et al. (2017) and Montgomery et al. (2018)
	Chemical treatments of fecal matter	2	Appiah–Effah et al. (2020)
	No defecation near/in the water body	3	Deoshatwar et al. (2022)
Hand wash	Ensuring proper hand washing principles	1	Hutin et al. (2003) and Rabbani and Greenough (1999)
	Washing hands before and after cooking and eating	2	Deoshatwar et al. (2022) and Rabbani and Greenough (1999)
	Washing hands around sick patients	3	Snow (1856)
Eating habits	Thoroughly cooking and preparing food	1	Rabbani and Greenough (1999)
	Avoiding seafood during disease outbreaks	2	Rabbani and Greenough (1999)
	Encouraging peeled vegetables and fruits	3	Rabbani and Greenough (1999) and Taylor et al. (2015)
Oral cholera vaccine	Before exposure (7–10 days before infection)	1	Song et al. (2021)

Finally, the uncertainty in the failure of predictive intelligence derived from mathematical algorithms for human infectious diseases remains a concern since technological integration of earth observations based on geophysical processes in disease models is a relatively new disciplinary domain. However, despite uncertainty, the false positive (no cholera outbreak when algorithms predicted conditions ripe for an outbreak) does provide a secondary benefit. This opportunity necessitates access to safe drinking water that can lead to human well‐being and health in the war‐torn regions of Ukraine. It is possible to save lives in the cholera‐risk regions of Ukraine if actions are taken now.

## Conflict of Interest

The authors declare no conflicts of interest relevant to this study.

## Data Availability

All the data are available in public domain from NASA web servers and at https://vibrio-prediction-ufl.hub.arcgis.com/.
